# Identification of Inhibitor Binding Site in Human Sirtuin 2 Using Molecular Docking and Dynamics Simulations

**DOI:** 10.1371/journal.pone.0051429

**Published:** 2013-01-28

**Authors:** Sugunadevi Sakkiah, Mahreen Arooj, Manian Rajesh Kumar, Soo Hyun Eom, Keun Woo Lee

**Affiliations:** 1 School of Life Sciences, Gwangju Institute of Science and Technology, Gwangju, South Korea; 2 Steitz Center for Structural Biology, Gwangju Institute of Science and Technology, Gwangju, South Korea; 3 Department of Chemistry and Institute of Basic Science, Chonnam National University, Gwangju, South Korea; 4 Division of Applied Life Science, Systems and Synthetic Agrobiotech Center, Plant Molecular Biology and Biotechnology Research Center, Research Institute of Natural Science, Gyeongsang National University, Jinju, South Korea; Bioinformatics Institute, Singapore

## Abstract

The ability to identify the site of a protein that can bind with high affinity to small, drug-like compounds has been an important goal in drug design. Sirtuin 2 (SIRT2), histone deacetylase protein family, plays a central role in the regulation of various pathways. Hence, identification of drug for SIRT2 has attracted great interest in the drug discovery community. To elucidate the molecular basis of the small molecules interactions to inhibit the SIRT2 function we employed the molecular docking, molecular dynamics simulations, and the molecular mechanism Poisson-Boltzmann/surface area (MM-PBSA) calculations. Five well know inhibitors such as suramin, mol-6, sirtinol, 67, and nf675 were selected to establish the nature of the binding mode of the inhibitors in the SIRT2 active site. The molecular docking and dynamics simulations results revealed that the hydrogen bonds between Arg97 and Gln167 are crucial to inhibit the function of SIRT2. In addition, the MM-PBSA calculations revealed that binding of inhibitors to SIRT2 is mainly driven by van der Waals/non-polar interactions. Although the five inhibitors are very different in structure, shape, and electrostatic potential, they are able to fit in the same binding pocket. These findings from this study provide insights to elucidate the binding pattern of SIRT2 inhibitors and help in the rational structure-based design of novel SIRT2 inhibitors with improved potency and better resistance profile.

## Introduction

The Sir2 (silence information regulator 2) or sirtuin family of class III deaceatylases differs from class I and II histone deacetylases (HDACs) by their sequences and structure [1]. Sirtuins are evolutionarily conserved NAD^+^-dependent protein deacetylases and adenosine diphosphate (ADP)-ribosylases. Seven NAD^+^-dependent HDAC proteins were recognized in mammalians, SIRT1-7 differs in the subcellular localization, substrate specificities, and functions. Sirtuin catalyze the deacetylation of lysine residues on histones and various proteins, resulting in a deacetylated product as nicotinamide, and O-acetyl-ADP-ribose [2]–[5].

**Figure 1:**
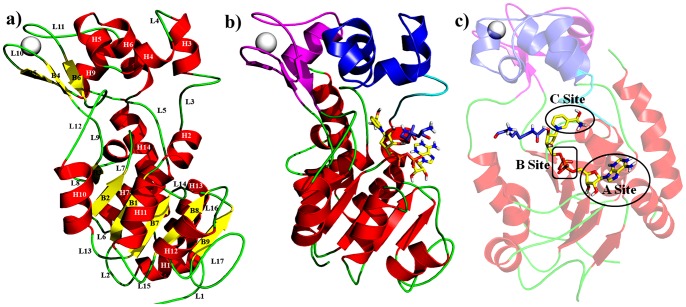
Structural details of human Sirtuin 2.

The catalytic core of sirtuins, conserved from bacteria to human with variable N- and C-terminals, contains approximately 250 amino acids. The catalytic domain consists of a large typical Rossmann fold or the classic pyridine dinucleotide binding fold, and a small domain composed of residues from two insertions within the Rossman fold, one comprising a zinc-binding module that contains a structural zinc atom coordinated by 4 invariant cysteine's, and the other forming a helical module that includes a flexible loop. The protein and NAD^+^ co-substrates bind in a cleft between the large and small domains. The cofactor–binding pocket can be divided into 3 regions: A-Site: binding of adenine ribose moiety of NAD^+^, B-Site: Nicotinamide ribose binding moiety and C-Site: located deep inside the pocket and contains the catalytic center Fig. 1 [6].

The members of Sirtuin family play an important role in biological processes, such as life span regulation [7]–[11], fat metabolization in human cells [12], insulin secretion [13], cellular response to stress [11], [14], [15], axonal degeneration [16], basal transcription factor activity [17], regulationg enzyme activity [18], rDNA recombination [19]–[21], and switching between morphological states in *Candida* [22], and apoptosis [23]–[25], there has been substantial progress in uncovering the chemical and structural details of these fascinating enzymes.

Salermide, one of the Sirtuin 2 (SIRT2) inhibitor, inhibits its function via depression of pro-apoptotic genes and p53 etc [26]. AcK40 on a-tubulin is a substrate for SIRT2; an important target for both cell division and cellular motor functions [27], [28] and tubulin dynamics is dependent on the acetylation level [29]. The anti-cancer activity of SIRT2 inhibitors was shown to occur simultaneous to cancer cell inhibition [30] but whether this is symptom or causes remains to be established. SIRT2 was shown to mediate apoptosis also in part via FOXOs and increased expression of Bim [31] and its inhibition associated with neuroprotection in a model of Parkinson's diseases [32]–[34].

All the inhibitors share a common binding site; the nature of specific interactions within the pocket is rather unique to each of the molecules. Moreover, the extent of binding derived from each of the pocket residues involved in ligand interaction is different for each of the inhibitors. The binding modes developed using mutation data are further refined and validated using available structure-activity relationship information. The sub-pockets are fully characterized for shape and electrostatic nature, and a refined pharmacophore model for lead identification can be generated with ease. This fully mapped binding pocket for SIRT2 has been effectively used as a structure-based design tool in the lead optimization of SIRT2 inhibitors.

In this contribution, molecular docking, molecular dynamics simulations, and quantum chemical calculations were done for 5 well know SIRT2 inhibitors, to explore their recognition by some amino acids involved in the SIRT2binding site, special attention to the electronic effects, which are related to the HOMO-LUMO energies of the compounds were taken into account.

## Materials and Methods

### Molecular docking

SIRT2 is one of the emerging drug targets in the drug discovery field. Unfortunately so far the SIRT2-complex structure was not crystallized. Hence, we applied the molecular docking study which is one of the computational tools to predict the interactions between the two or more molecules such as protein-ligand, protein-protein, and protein-DNA, to construct the suitable complex structures for SIRT2. In order to gain the insight into the recognition between the SIRT2 and inhibitors, molecular docking simulations were done on the 3-D structure of SIRT2 since they represent the pharmacology target for the development of new drugs to treat various diseases.

### GOLD

**Figure 2:**
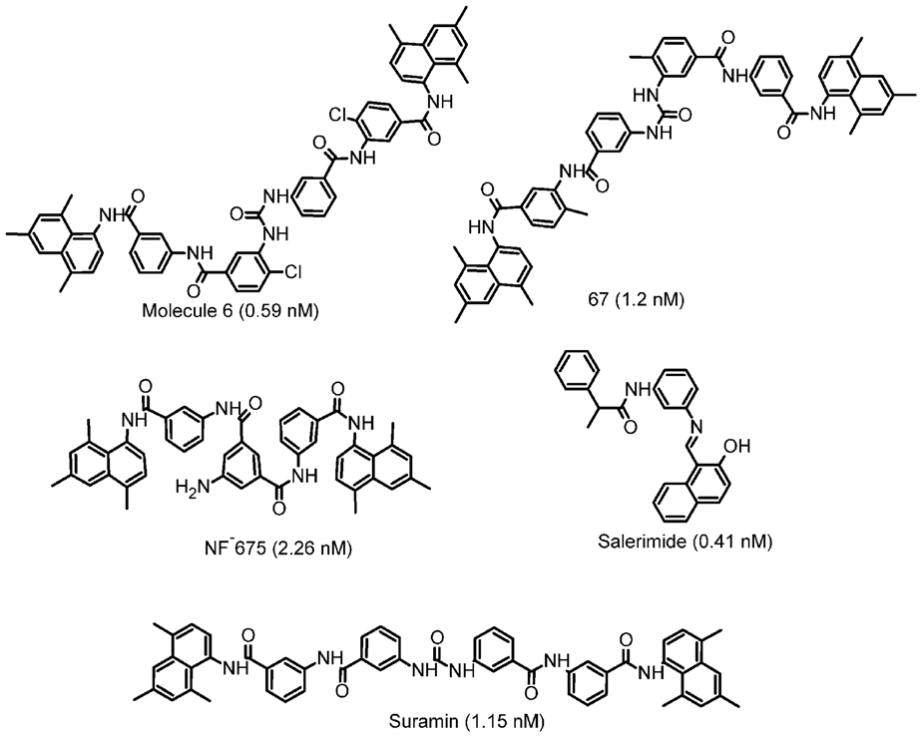
The 2D structure of the 5 structurally diverse SIRT2 inhibitors and its IC50 nM values are shown in the bracket.

To obtain the suitable starting structures of SIRT2-complex (with inhibitors) for MD simulations, GOLD v 5.1 (Genetic Optimization for Ligand Docking) molecular docking was performed to explore the probable active site of SIRT2. GOLD uses a genetic algorithm (GA) for docking flexible ligands into protein binding sites to explore the full range of ligand conformational flexibility with partial protein flexibility. The Apo form of SIRT2 (PDB ID: 1J8F) was taken from the RCSB protein data bank. We selected the five structurally diverse SIRT2 inhibitors (Fig. 2) with representative good biological activity from various literatures [26], [30], [35]. It is very important to define the proper binding site for the small molecules. In this work, we specified the approximate center of the binding site and took all atoms that lie within a specified radius of 8 Å from Gln167:O. Only those atoms are included in the binding site which was considered during docking process. The binding site definition should therefore be large enough to contain any possible binding mode of the ligand, and include all atoms or residues that might be involved in ligand binding. The standard default settings such as population size 100, selection pressure 1.1, niche size 2, migrate 10, crossover 95, number of operations 100,000, number of dockings 10 were adapted for docking process. The Gold Score was opted to select the best docked conformations of the SIRT2 inhibitors in the active site. The annealing parameters of van der Waals and H-bond interactions were considered within 4.0 and 2.5 Å, respectively. Empirical parameters used in the fitness function (hydrogen bond energies, atom radii and polariabilities, torsion potentials, hydrogen bond directionalities, etc.) are taken from the GOLD parameter file. These parameters are independent of the scoring function being used. The best SIRT2 complexes are selected based on the gold fitness score and the critical interactions reported in the literatures.

### Identification of putative binding pocket using Molecular dynamics simulations

Molecular dynamics simulations methods involve the calculation of solutions to Newton's equations of motions. Based on the docking results, five best SIRT2 complexes were selected to conduct the MD simulations using the GROMACS v4.5.5 with Amber03 force field. AM1-BCC charges were calculated for inhibitors using Antechamber software. The cysteine residues which interact with zinc ions were chosen as negatively charged cysteine (CYM). Each system was inserted in the layer of TIP3P water box surface at least 1 nm away from the SIRT2 complex. The entire systems were neutralized by adding Na^+^ counter ions by replacing solvent molecules. The systems were subjected to 10,000 steps steepest descent energy minimization. Then the protein backbone was frozen and the solvent molecules with counter ions were allowed to move during a 100 ps position restrained MD run. The production run was 20 ns. All simulations were run under periodic boundary conditions using Berendsen's coupling algorithm for maintaining the temperature (300 K) and the pressure constant (1 bar). The SHAKE algorithm with a tolerance of 10–15 Å was applied to fix all bonds containing hydrogen atoms. The electrostatic interactions were calculated by using the Particle-mesh Ewald (PME) algorithm, with an interpolation order of 4 and a grid spacing of 0.1 nm. The van der Waals forces were treated by using a cutoff of 10 Å and the coordinates were stored every 1 ps. All the preliminary analyses like root mean square deviation (RMSD), root mean square fluctuation (RMSF), and the secondary structural analyses were carried out by GROMACS analysis programs.

### Identification of putative binding pocket using GOLD

The representative structures of each SIRT2-complex from MD simulations were selected as receptors. The same five well known SIRT2 inhibitors were re-docked in the adjusted active site of SIRT2 applying similar parameters from GOLD to refine the putative binding site as well to define the perfect orientation of the inhibitors. The representative structure gives a clear insight of the structural changes due to the inhibitor binding as well as much helpful to suggest the interaction between the SIRT2 and its inhibitors.

### Computation of Binding energy

The binding energy (i.e., van der Waals and electrostatic energies) was calculated for these complexes using Calculate binding energies protocol/DS. The free energy of binding for a receptor-ligand complex can be calculated from the free energies of the complex, the receptor, and the ligand. Using CHARMm based and implicit solvation methods it is possible to estimate these free energies and thus calculated as estimate for the overall binding free energy. The binding free energy is calculated using the following equation:
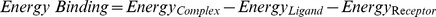
The following three steps are performed during free binding energy calculations. 1. In situ ligand minimization was performed to remove the van der Waals clashes of ligand prior to calculating the binding energy and conformational entropy. 2. Calculate ligand conformational entropy, and 3. Binding energy.

### Quantum mechanics/molecular mechanics calculations: Frontier molecular orbital energy calculated using DFT method

QM/MM calculations were performed to obtain the HOMO, LUMO, energy gap (Ehomo-Elumo) of the reactive complex formed by inhibitors and the SIRT2. The minimized energy structures of the compounds and some selected SIRT2 residues were obtained by means of DFT calculations at the B3LYP/6-31+(d,p) level, with the aid of the Gaussian 03 package [36].

Density functional theory (DFT) is presently the most successful approach to compute the electronic structure of matter [37]. The popular quantum mechanical descriptors such as HOMO and LUMO play a major role in governing many chemical reactions [38], [39]. DFT has been used to predict the stability of the charge transfer or to study the reaction mechanisms responsible for the antioxidant activity. Recently, there is growing evidence that DFT provides an accurate description of the electronic and structural properties of small molecules by computing the electronic structure of matter. The main purpose to utilize the DFT approach is to theoretically characterize the electronic properties and structure-property to correlate with the SIRT2 inhibitors. The energy of the SIRT2 inhibitors were calculated using *Calculate Energy module* by combining the quantum mechanics (QM) and molecular mechanics (MM) force-field. It calculates the QM-MM single point energies and geometry optimization minimizations using Dmol^3^ as the quantum server with CHARMm force-field. This protocol simulates the systems by dividing the input into two regions, central and outer regions which was treated by quantum and molecular mechanics methods as well as it calculates the electronic orbital properties for a molecules such as HOMO and LUMO. The optimized molecules were used to calculate the HOMO and LUMO energy values.

### Molecular Electrostatic potential calculations

The formatted check point file of the compounds are generated by the geometric optimization computation were used as input for CUBEGEN program interface with Gaussian 03 program to compute the MEP.

## Results and Discussion

Currently, one of the most challenging problems in computational chemistry is to accurately predict the binding mode of the small ligands in the active site of proteins. To understand the interactions between SIRT2 and its inhibitors, five well know SIRT2 inhibitors were selected from the literatures. Initially, molecular docking calculation was performed using the 5 inhibitors to dock in the NAD^+^ binding site of SIRT2. The inhibitors with the most favorable free binding energies and reasonable orientations were selected as the optimal docked conformations. To acquire the further binding mode of ligand-SIRT2 complex, we took the flexibility of the protein into consideration and selected the optimal docked conformations of 5 best complexes to preform MD simulations.

### Initial orientation of the inhibitors in SIRT2 active site

The ligand which shows the greatest interaction with SIRT2 was plotted using the LIGPLOT. Initially, the top 10 poses for each antagonist were saved based on the GOLD fitness score. The fitness score is taken as the negative of the sum of the component energy terms, such as protein-ligand hydrogen bond energy (external H-bond), protein-ligand van der Waals (vwd) energy (external vdw), ligand internal vdw energy (internal vdw), and ligand torsional strains energy (internal torsion) so that larger fitness scores are better. The gold fitness score value of suramin, salerimide, 67, Mol-6, and NF-675 are 65.70, 53.29, 66.98, 47.99, and 40.90, respectively (Table 1). The hydrogen bond interactions are manually analyzed using the Discovery Studio v3.1. Suramin shows hydrogen bond interactions with Ala85, Ser88, Thr89, Arg97, Gln167, His 187, and Asp170. All the inhibitors showed hydrogen bond interactions with Gln167. Salerimide shows hydrogen bond interactions with Ala85, Gln167, Ile169, and His187. 67 shows hydrogen bond interactions with Ala85, Thr89, Asp95, Phe96, Arg97, and Gln167. Mol-6 shows hydrogen bond interactions with Asp95, Phe96, Gln167, His187, and Gln267. NF-675 shows hydrogen bond interactions with Gln167, ASn168, Gly261, Thr262, and Gln267. The SIRT1-antagonist complexes which show a good fitness as well as produce the important hydrogen bond interactions with the active site residues were selected and subjected into the molecular dynamics simulations to refine the side-chain orientations as well as to characterize possible conformational rearrangements in the protein structure which could have favorable effects on ligand binding.

**Table 1:** Initial docking of five different inhibitors in the active site of SIRT2.

Inhibitor	1J8F_Interaction	Gold Fitness Score
Suramin	Ala85,Ser88,Thr89, Arg97,**Gln167**,His187, Asp170	65.70
Salermide	Ala85,**Gln167**, Ile169, His187	53.29
67	Ala85, Thr89, Asp95, Phe96, Arg97, **Gln167**	66.98
Mol-6	Asp95,Phe96, **Gln167**, His187, Gln267	47.99
NF-675	**Gln167**,Asn168, Gly261,Thr262, Gln267	40.89

### Identification of putative binding pocket using molecular dynamics simulations

#### Stability of the SIRT2 complexes

**Figure 3:**
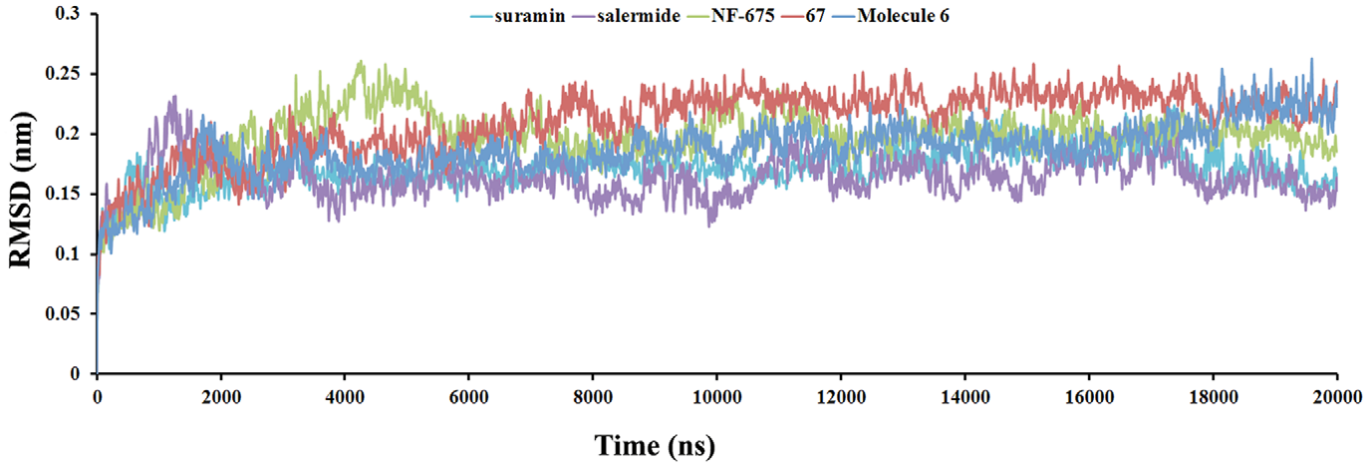
The stability of the five different systems where calculate based on the root mean square deviation of Cα atoms.

Totally five Sirtuin complex systems such as SIRT2-suramin, SIRT2-salermide, SIRT2-67, SIRT2-mol6, and SIRT2-nf675 were prepared for 20 ns MD simulations. To gauge whether the MD simulations were stable and converged, energetic and structural properties were monitored during the course of MD simulation. During the MD simulations the stability and the fluctuation of the complex structures were evaluated by the time dependent evaluation of Cα root mean square deviation (RMSD) and root mean square fluctuation (RMSF) and convergence of energies, temperatures, and pressure of systems indicate well-behaved systems. The RMSD was calculated during 20 ns production phase using the respective initial minimized structure as the reference structure. The averaged RMSD values during the last 10 ns for SIRT2-complexes are 0.166 nm, 0.178 nm, 0.228 nm, 0.199 nm, and 0.202 nm for salerimide, suramin, 67, mol6, and nf675, respectively. The RMSDs between the Cα atoms of the structures obtained during the trajectories are shown in Fig. 3. The hydrogen bonds between the ligand and the critical amino acids are stable in the active site of SIRT2 suggesting the overall stable structure after approximately 10 ns simulations. Secondary structural analysis was carried out to measure the stability of the simulations. These analysis shows that the complex secondary structures are persist throughout the simulation time.

### Identification of the putative binding pocket

To identify the putative binding pocket for SIRT2 inhibitors, we compared all the five representative structures from the MD simulations. In order to find the refined binding pocket of the antagonist in SIRT2, we re-docked all the inhibitors using the representative as a receptor from each system. The putative binding pocket was selected around 8 Å vicinity of the critical residue Gln167 which is one of the crucial residues in the representative SIRT2 complex from MD simulations.

### GOLD Molecular Docking using Representative Structure as a receptor

**Figure 4:**
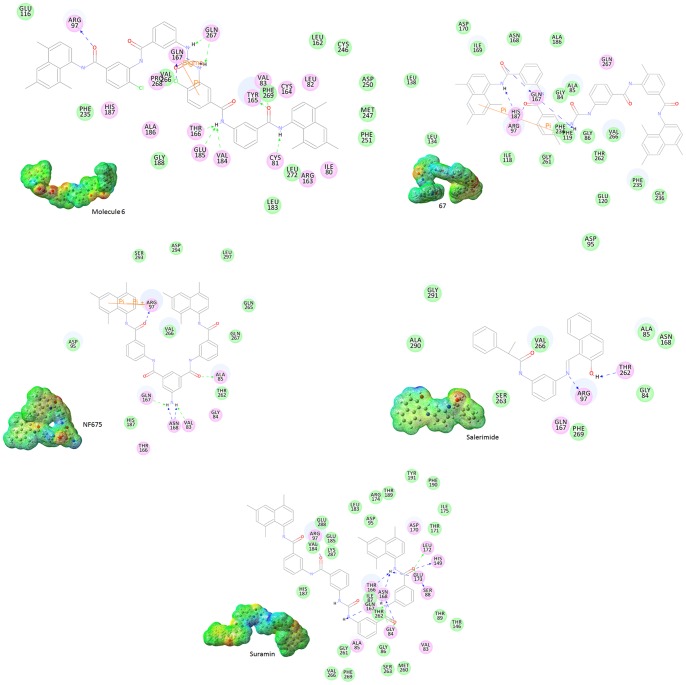
Molecular docking. Residue Interaction based on color code: Magenta-Electrostatic Interaction; Green – Van der Waals Interactions. The blue dashed arrow represent that hydrogen bond interactions with side chain amino acid, the green dashed arrow indicates hydrogen bond interactions with the main chain amino acids.

We docked the 5 well know inhibitors in the active site of the five different representative structures selected from MD. Suramin representative structure shows a gold fitness scores of 70.82, 51.97, 70.88, 65.52, and 72.79 for suramin, salerimide, Mol-6, 67, and NF675, respectively. Salermide representative structure shows a gold fitness scores of 77.07, 55.75, 76.47, 86.01, and 81.34 for surnamin, salerimide, Mol-6, 67, and NF675, respectively. 67 representative structure shows a gold fitness scores of 76.98, 75.39, 80.49, 69.90, and 79.97 for surnamin, salerimide, Mol-6, 67, and NF675, respectively. Suramin representative structure shows a gold fitness scores of 70.82, 51.97, 70.88, 65.52, and 72.79 for suramin, salerimide, Mol-6, 67, and NF675, respectively. Mol-6 representative structure shows a gold fitness scores of 57.44, 54.70, 63.30, 46.34, and 51.08 for suramin, salerimide, Mol-6, 67, and NF675, respectively. NF-675 representative structure shows a gold fitness score of 81.45, 67.42, 90.18, 82.01, and 81.34 for suramin, salerimide, Mol-6, 67, and NF675, respectively (Table 2). The docking results showed a highest gold fitness score for Mol-6 except salerimide-complex representative structure. Manually the hydrogen bond interactions were checked using DS (Table 3). All the five inhibitors show the common hydrogen bond interactions with Asp97 and Gln167 (Fig. 4). *Tervo et. al.* [40], and *Sakkiah et. al.* [41], reported that Gln167 is one of the potential hydrogen bond donor, similarly our docking results have shown a hydrogen bond interactions with Gln167 which reveal that the interactions between the Gln167 and the small molecules will be crucial to inhibit the SIR2 activity.

**Table 2:** Gold Fitness Score for representative docking structures.

Inhibitor/Receptor	Suramin	Salermide	67	Mol-6	NF-675
Suramin	70.82	77.07	76.98	57.44	81.45
Salermide	51.97	55.75	75.39	54.70	67.42
67	70.88	76.47	80.49	63.30	90.18
Mol-6	65.52	86.01	69.90	46.34	82.01
NF-675	72.79	81.14	79.97	51.08	81.34

**Table 3:** Hydrogen Bond Analysis.

Inhibitor/Receptor	Suramin	Salermide	67	Mol-6	NF-675
Suramin	Asp95,**Asp97**, Ser98,**Gln167**, Thr262,Gln267	Ala85,**Arg97**, **Gln167**,Asn168	**Arg97**,His187, Phe235, Ser238	Gly84,**Arg97**, His149,**Gln167**,Asn168,Leu172,Glu173	**Arg97,Gln167**,Gln267
Salermide	**Arg97,Gln167**, Thr262	Ala85,Gly86, **Gln167**,Asn286	**Arg97,Gln167**, His187	**Arg97,Gln167**, Thr262	**Arg97,Gln167**,Asn168, Ile169, Thr262
67	**Arg97**,Glu116, **Gln167**,Thr262	Ala85,**Arg97**, **Gln167**,Asn168,Thr262, Ser263	**Arg97**,His187, Ser238	**Arg97,Gln167**, His187	**Arg97,Gln167**,Asp294
Mol-6	Ala85,Asp95, **Arg97**,Ser98, **Gln167**,Thr262, Gln267	Ala85,**Arg97**, **Gln167**,Thr262, Ser263	**Arg97**,His187, Phe234,Phe235, Ser238	Val83, **Arg97**, Tyr165,Thr166, **Gln167**,Val184,Glu185,His187, Gln267	**Arg97,Gln167**,Thr262, Gln267
NF-675	**Arg97,Gln167**, Thr262,Gln267	Ala85,**Gln167**, Asn168,Asp170, Ser263	**Arg97**, Glu116, Phe119 His187,	Val83, Ala85, **Arg97,Gln167**, Asn168	**Arg97, Gln167**, Ser263

### Computation of Binding energy

Table 4 shows the binding energy of the five inhibitors with each representative receptor. Comparing all the different orientations of the inhibitors in SIRT2, Mol-6 active site shows good interactions and the binding affinity for 5 well known inhibitors. Hence, among these 5 receptors, we selected Mol-6 receptors and the inhibitors for further studies. Arg97 and Gln167 play an important role in hydrogen bond interactions and give a strong binding affinity for the inhibitors.

**Table 4:** Binding energy for five different inhibitors in the active site of SIRT2.

Inhibitor/Receptor	Suramin	Salermide	67	Mol-6	NF-675
Suramin	−198.70	−522.84	−212.49	**−353.63**	−417.31
Salermide	−123.59	−223.57	−151.65	**−237.09**	−345.52
67	−150.86	−311.50	−204.92	**−369.02**	−353.09
Mol-6	−159.80	−673.51	−235.20	**−356.31**	−443.76
NF-675	−140.78	−413.73	−154.76	**−259.54**	−385.08

### Molecular electrostatic potential

**Figure 5:**
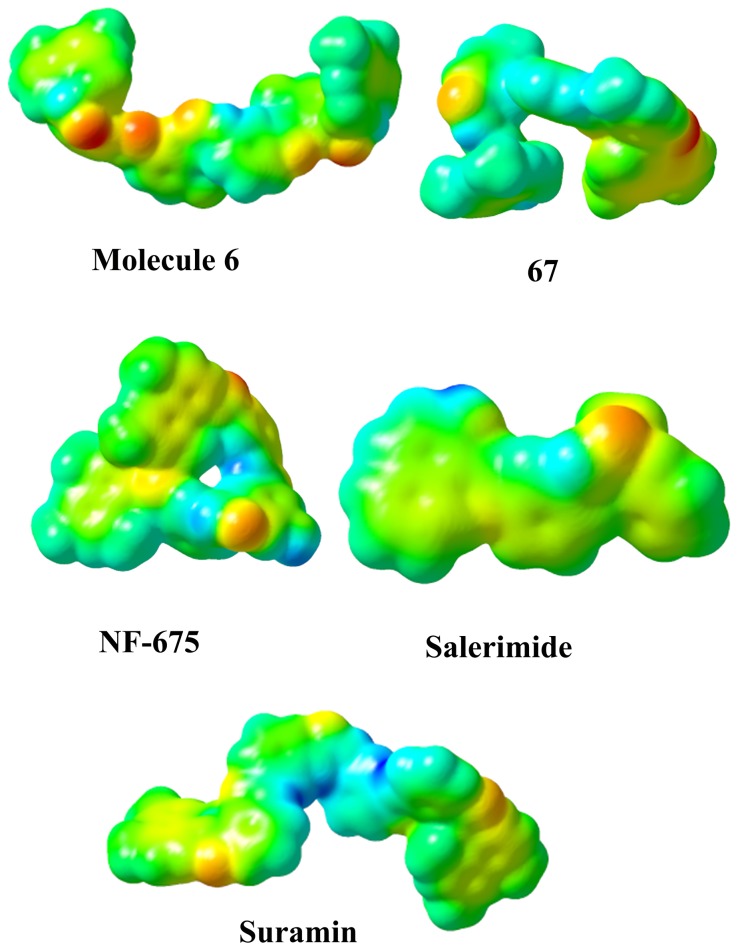
The electrostatic potential energy for five different SIRT2 inhibitors.

Suramin, salermide, 67, mol6, and nf675 have shared the same binding pocket, although show a different in shape and electrostatic potential. The conformational analysis showed that the conformational space accessed by these compounds is very different. The best pose (conformation) from molecular docking study was selected to generate the electrostatic potential maps. The SIRT2 receptor is able to accommodate the structurally and electrostatically diverse antagonists by using a critical set of interactions with each ligand. The molecular electrostatic potential was applied to interpret and predict the reactive behavior of the electrophilic and nucleophilic reactions. The MEP plays a key role in the initial step of bioactive conformation explaining the interactions between the ligand-receptor. The different values of the electrostatic potential at the surface are represented by different colors; red represents regions of most negative electrostatic potential, blue represents regions of most positive electrostatic and green represents regions of moderate potential. Potential increases in the order red<orange<yellow<green<blue. Red, green, and blue color indicates the high accumulation of the negative charge, neutral region, and the positively charge region, respectively. The MEP isopotential surfaces was produced and superimposed onto the total energy density surface (0.0004 e/au3). The 3D MEP surfaces plotted for SIRT2 inhibitors were shown in Fig. 5. The MESP plotted for different five inhibitors have showed the most electronegative potential region (red color) over the oxygen atom in the peptide bonds. The most interesting thing is that the oxygen atom in the peptide bond which shows the higher negative charge was orientated adjacent to the Gln167 to make a strongest hydrogen bond interaction. The strong electrostatic interaction of the negative potential with key residues will enhance the inhibition effect substantially together with the orbital interaction through the exchange of energy. Docking result of these compounds also indicated that the hydrogen bind interaction with Gln167 is very crucial to inhibit SIRT2 function. Thus electrostatic potential of the inhibitors can play a significant role in the binding and interaction with sirutin2 together with orbital energies, and consequently influence the inhibition effect.

## Conclusions

Here we applied the most well-known computational techniques such as molecular docking, molecular dynamics simulation, and density functional analysis to explore the residues involved in the crucial molecular interaction with small molecules to inhibit the function of SIRT2. Due to the absence of complex SIRT2 structure in PDB, molecular docking studies were performed for the 5 well known inhibitors which were docked with the different scaffold in the SIRT2 active site. The best orientations of the 5 different inhibitors were selected and subjected into molecular dynamics simulation to refine the active site residues such as Gln167, Asp97, Ile167, Asp170, Asn168, and His187 in SIRT2 as well as to adjust the suitable orientation for the inhibitors. The MESP shows a clear view of the important electrostatic features of inhibitors to inhibit the activity of SIRT2. Therefore, the prediction of the SIRT2 druggable site and the identification of inhibitors binding provide the input for fragment-based combinatorial approaches which will be helpful to yield more potential lead-like molecules than the traditional high throughput screening.
